# Machine learning to assist risk-of-bias assessments in systematic reviews

**DOI:** 10.1093/ije/dyv306

**Published:** 2015-12-08

**Authors:** Louise AC Millard, Peter A Flach, Julian PT Higgins

**Affiliations:** ^1^ MRC Integrative Epidemiology Unit,; ^2^ School of Social and Community Medicine and; ^3^ Intelligent Systems Laboratory, University of Bristol, Bristol, UK

**Keywords:** Risk of bias, systematic review, text mining, machine learning

## Abstract

**Background:** Risk-of-bias assessments are now a standard component of systematic reviews. At present, reviewers need to manually identify relevant parts of research articles for a set of methodological elements that affect the risk of bias, in order to make a risk-of-bias judgement for each of these elements. We investigate the use of text mining methods to automate risk-of-bias assessments in systematic reviews. We aim to identify relevant sentences within the text of included articles, to rank articles by risk of bias and to reduce the number of risk-of-bias assessments that the reviewers need to perform by hand.

**Methods:** We use supervised machine learning to train two types of models, for each of the three risk-of-bias properties of sequence generation, allocation concealment and blinding. The first model predicts whether a sentence in a research article contains relevant information. The second model predicts a risk-of-bias value for each research article. We use logistic regression, where each independent variable is the frequency of a word in a sentence or article, respectively.

**Results:** We found that sentences can be successfully ranked by relevance with area under the receiver operating characteristic (ROC) curve (AUC) > 0.98. Articles can be ranked by risk of bias with AUC > 0.72. We estimate that more than 33% of articles can be assessed by just one reviewer, where two reviewers are normally required.

**Conclusions:** We show that text mining can be used to assist risk-of-bias assessments.

Key Messages

- Risk-of-bias assessments for systematic reviews are, at present, a manual process.
- Our results indicate that text mining can be used to automate elements of risk-of-bias assessments, in order to assist systematic reviewers.
- We show that text mining can be used to identify information pertinent to risk of bias in research articles describing clinical trials.
- We also show that text mining can be used to predict the level of risk of bias from the full-text content of research articles describing clinical trials.

## Introduction

Systematic reviews combine evidence from multiple studies to answer a research question more comprehensively than is possible from an individual study. When combining the evidence from several studies, it is important to consider the risk of bias in each study. Results of clinical trials may be biased if the study methods are not adequate. For example, allocation of incoming participants to groups should be concealed during the randomization process. ^1^^,^^2^ Assessing risk of bias in clinical trials typically involves extraction of information sufficient to assign a judgement on the adequacy of each methodological property affecting risk of bias. ^3^

Thorough systematic reviews are time consuming, often lasting up to 3 years and requiring two reviewers to assess each research article to minimize errors. One study estimated that 80% of risk-of-bias assessments took between 10 and 60 min to perform. ^4^ Furthermore, risk-of-bias judgements are imperfect. Studies have shown that reviewers often report different levels of risk of bias for the same studies. ^5–9^ This may happen, for instance, if a reviewer misses key sentences. ^8^

Automating aspects of risk-of-bias assessments has the potential both to reduce the time required to perform a review and to reduce human error and subjectivity in the reviewing process. Identifying relevant sentences and predicting a risk-of-bias assignment from text in articles are tasks that text mining methods can potentially perform automatically. Several studies have examined text mining methods to predict elements of studies for systematic reviews, ^10–22^ but we know of only one research group that has investigated automating the prediction of risk-of-bias properties. ^23–25^ An additional potential benefit of automating aspects of risk-of-bias assessments is the ability to identify the high quality studies (with low risk of bias) early in the process so that these can be prepared for inclusion in the analyses. This has particular appeal in the context of rapid reviews. ^26^^,^^27^

In this work we investigate the use of text mining methods to predict risk-of-bias properties, in order to assist systematic reviewers with their work. We focus on the following three risk-of-bias elements: the method of random sequence generation; the use of concealment methods when participants are allocated to the study groups; and the method of blinding of participants and personnel. These we refer to as the properties sequence generation, allocation concealment and blinding, respectively. We work towards specific objectives of: (i) identifying relevant sentences within research articles; (ii) ranking articles by risk of bias; and (iii) reducing the number of assessments the reviewers need to perform by hand. We also compare performance when using the title and abstract from the PubMed database with the performance when using the full text article, to examine whether potential gains in performance from using the full text offset the cost of its retrieval.

## Dataset

**Figure 1.**
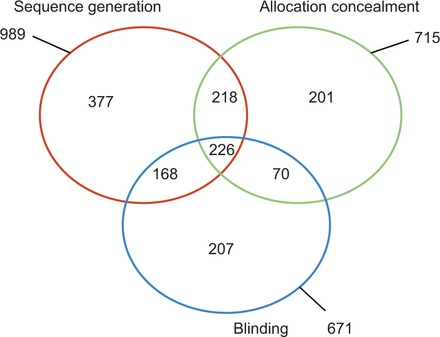
Venn diagram of data in our dataset, showing the number of articles with a value for each property.

Our dataset consists of a set of 1467 full-text articles, each with a value assigned for at least one of the three risk of bias properties (sequence generation, allocation concealment and blinding), where the value is supported by some part of the text in the article (summarized in Figure 1 and Table 1 ). These risk-of-bias values are either low or not-low, denoting whether a particular property has a low risk of causing bias. Our dataset also includes a binary label attached to sentences, denoting whether they contain relevant information with respect to a risk-of-bias property. Each sentence is relevant or not-relevant or is unlabelled. We also have the title and abstract text of each research article in our dataset, retrieved from the PubMed database. The full text was extracted from the research articles using the Adobe PDFBox text extraction tool (version 1.8.6). Sentence segmentation was performed with the PTBTokenizer of the Stanford CoreNLP Java package (version 3.4.1).

**Table 1.** Number of studies with a value of each property in our dataset, and in the original data files of the Cochrane risk of bias tool

		Our dataset		Original data source	
Property	Property value	Number	Proportion	Number	Proportion
*blind*	low	361	0.538	7120	0.415
	not-low	310	0.462	10 021	0.585
	total	671		17 141	
*seq-gen*	low	495	0.501	6846	0.408
	not-low	494	0.500	9942	0.592
	total	989		16 788	
*alloc-conc*	low	327	0.457	6057	0.338
	not-low	388	0.543	11 840	0.662
	total	715		17 897	

We constructed this dataset using data collected from the Cochrane Database of Systematic Reviews and specifically from the Cochrane risk-of-bias assessment tool. ^3^ The data from this tool include references of articles that the reviewer assessed for each clinical trial. Each trial also has a low, high or unclear value assigned to each risk-of-bias property. A value of low for blinding, for instance, means that blinding was adequately performed in this study such that the risk of bias is low. These judgements are supported by text descriptions, often including direct quotations from articles or a comment that no information was found in the article.

**Figure 2.**
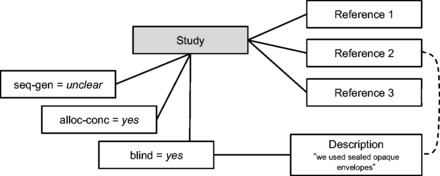
Data from the Cochrane risk of bias tool. Dotted line represents a relationship that we need to infer.

**Figure 3.**
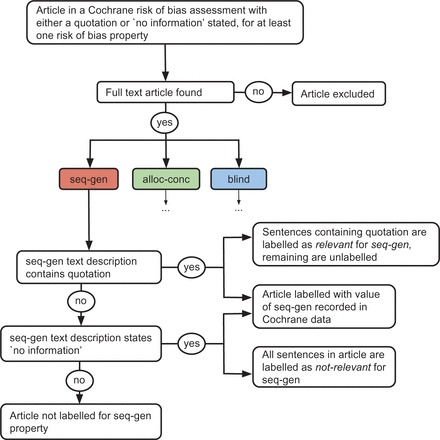
Flow diagram illustrating dataset creation process, from Cochrane risk-of-bias data to labelled dataset.

The Cochrane data do not specify which articles contain the information that informed the risk-of-bias judgement. We use the text descriptions to infer this. First, articles containing quoted text contain information used to make the judgement. For instance, Figure 2 shows an example where a study has a quotation for the blinding property, which is found in the article content of reference 2. We then infer that the blinding judgement was made using information in this particular article. Articles where ‘no information’ was stated do not contain any information, and we can infer that the lack of information is the reason for this choice of label value. For instance, an article may have the label unclear for blinding and ‘no information’ in the text description because all research articles referenced for this study in this review have been found to contain no relevant text, such that an assignment for the property value to low or high could not be given. We only include articles in our dataset when either no information is stated, or a quotation is found in the article text. The flow diagram in Figure 3 illustrates our dataset creation process.

We combine the high and unclear labels in the Cochrane data to give a binary variable with values low and not-low. We justify this on the basis that a reviewer generally wants to identify the high quality studies, such that the articles of high and unclear risk of bias can be grouped together.

We use the quotations and ‘no information’ statements to label sentences as relevant or not-relevant. A sentence is relevant if it contains a quotation supplied for this article in the Cochrane risk of bias assessment. A sentence is not-relevant if it is within an article associated with a study where ‘no information’ was stated. Otherwise, a sentence is unlabelled. We cannot determine the label of the unlabelled subset because, when a reviewer provides a quotation during a risk-of-bias assessment, they are likely to choose only exemplary text rather than to include quotations for all relevant text in an article. Hence, these unlabelled sentences may contain relevant information. Further details of the dataset are provided in supplementary information S1 (available as Supplementary data at *IJE* online).

## Statistical and Machine Learning Methods

**Figure 4.**
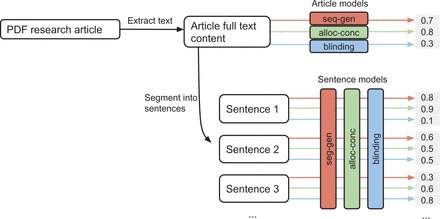
Flow diagram illustrating process from PDF article to predicted scores. The article model predicts a score denoting the risk of bias, and the sentence model predicts a score denoting the relevance of a sentence, for each risk of bias property.

We use two types of models to make predictions from the article text independently of each other. The first, used for objective 1, predicts the relevance of each sentence of an article using the words it contains. The second, used for objectives 2 and 3, predicts the risk of bias in a study from the words contained in an article. A flow diagram illustrating the process from PDF articles to model predictions is shown in Figure 4 .

We use logistic regression to create the sentence-level and article-level models. We did not use regularization—see supplementary material S2 (available as Supplementary data at *IJE* online) for an explanation of this choice. In line with the domain-based nature of a risk-of- bias assessment, we implement this individually for each risk-of-bias property: sequence generation, allocation concealment and blinding. The dependent variable for a sentence-level model is the binary variable with values relevant or not-relevant, indicating whether a sentence contains information relevant to a particular risk-of-bias property. The dependent variable for an article-level model is the binary variable with values low or not-low, describing whether a particular property has a low risk of causing bias, as described by the contents of the article. Each independent variable is the absolute number of occurrences of a word in a sentence or article respectively (known as a ‘bag of words’ representation with unigram features).

Logistic regression provides predictions in terms of a score that denotes the likelihood of each particular label. We use logistic regression as it has the following attractive features. First, the parameters of logistic regression have a clear interpretation. Given the logistic model *y = 1/(1 + e ^(^^β^^1^^ ^^×^^ ^^x1^^ ^^+^^ ^^β^^0^^)^ )* , a one-unit increase in the independent variable *x _1_* corresponds to a *β _1_* change in the log odds of *y.* Second, logistic regression is known to produce scores that are well calibrated. Scores are calibrated if, for example, given a set of articles that all have a score of 0.8, we can expect 80% of these articles to have a label of low (assuming a high score denotes more likely to be low). This means that we can use these scores as probabilities that an article (or sentence) belongs to a particular class. ^28^^,^^29^

We perform the following pre-processing of the features, commonly performed in text mining tasks. We convert the terms to lower case such that, for instance, the words ‘random’ and ‘Random’ correspond to a single parameter in the model. We perform word-stemming using Porter’s algorithm. We remove common words, known as stop words, from the set of features as these are unlikely to be predictive and vastly increase the number of features. We remove words that occur fewer than ten times in the dataset and words of one or two characters in length. All remaining words are included in the models. We use the Weka machine learning package to perform the text pre-processing and estimate the parameters of the logistic regression models with stochastic gradient descent (SGD in Weka, with sentence-level parameters, learning rate: 0.001, lambda: 0, epochs: 2000; and article-level parameters, learning rate: 0.0001, lambda: 0, epochs: 4000). The number of examples is far higher for the sentence-level learning compared with the article-level learning and so we use fewer epochs for the sentence-level learning such that the time taken to run the analysis remains practical, and a larger learning rate compensates for the reduced number of epochs.

As is common practice in machine learning, we evaluate model performance using 10-fold cross validation where we train the model (i.e. estimate its parameters) using 90% of the data, and test it on the other 10%; and this is repeated 10 times with different 90/10 splits of the dataset (where each split is called a fold). This avoids over-optimistic estimates of performance, which can arise when the model is trained and tested on the same data. Our data are stratified such that each fold contains approximately equal numbers of positive and negative examples.

We use receiver operating characteristic (ROC) curves to assess the performance of the models visually. ^30^ These curves depict many evaluation metrics that can be used to evaluate model performance, for either classification or ranking tasks. For instance, the horizontal and vertical axes, the false and true positive rates (also known as 1-specificity and sensitivity, respectively) are two common metrics for evaluating classification performance.

### Methods for objective 1: identifying relevant sentences

This objective aims to rank sentences in order of relevance, for each risk-of-bias property. Each sentence in our dataset is one of three types, with respect to a particular risk-of-bias property: relevant, not-relevant or unlabelled. Using this labelling, there are two choices of dataset we can use to train the parameters of the logistic regression model. The first dataset uses the sentences known to be relevant as positive examples and not-relevant as negative examples, and does not include unlabelled sentences. Here we are trying to train a model that can distinguish relevant sentences from not-relevant sentences.We refer to this as the relevant/not labelling approach. The second option is to use the relevant sentences as the positive examples, and both the not-relevant and the unlabelled sentences as the negative examples (such that all sentences in the dataset are used). Here we would be trying to separate the relevant sentences from the rest. We refer to this as the relevant/rest labelling approach.

Our aim is to separate relevant sentences from not-relevant sentences, but it is not clear which dataset is preferable to train a model to do this. Whereas the relevant/not data clearly represent the relevant vs not-relevant notion more appropriately, the relevant/rest dataset has the advantage of a much larger sample size (see Table 2 ). Previous work by Marshall *et al.*^23^ used the relevant/rest labelling approach to separate relevant from not-relevant sentences, which assumes (as they note) that the unlabelled sentences are all not-relevant, and this is unlikely to be the case. The relevant/not labelling approach assumes that the subset of examples labelled as relevant and not-relevant are representative of the remaining sentences in the dataset, for which the labels are not known. We have no reason to believe that this assumption is not valid.

**Table 2.** Results for sentence level: predicting the relevance of each sentence with regards to a risk of bias property

		seq-gen	alloc-conc	blind
Number of sentences	Unlabelled	243 477	129 155	148 934
	Not-relevant	14 989	59 390	24 190
	Relevant	1667	514	1156
Mean AUC ^3^	A) relevant/rest	0.974 (0.008)	0.981 (0.009)	0.974 (0.007)
	B) relevant/not	0.987 (0.003)	0.986 (0.011)	0.991 (0.006)
	C) train relevant/rest, test relevant/not	0.978 (0.008)	0.983 (0.009)	0.980 (0.007)
*P* -value	A vs B ^1^	<0.001	0.229	<0.001
	B vs C ^2^	0.005	0.462	0.001

^1^ *P* -values using two-tailed unpaired t-test to compare the AUC values across the 10 folds of cross-validation (data are not matched).

^2^ *P* -values using two-tailed paired t-test to compare the AUC values across the 10 folds of cross-validation (data are matched).

^3^ Mean AUC across 10 folds of cross-validation.

We compared the use of the relevant/rest and relevant/not datasets for separating relevant sentences from not-relevant sentences. First, we train models with the relevant/rest dataset, and test these models also with the relevant/rest dataset (test A). We then train models with the relevant/not dataset and test these models also with the relevant/not dataset (test B). We compare the results using these two datasets, to give an indication of the predictive ability of each dataset. We also train models using relevant/rest sentences and test using the relevant/not dataset (test C). This allows us to assess how well a model trained with the relevant/rest dataset can separate the relevant sentences from the not-relevant sentences, even though the unlabelled data are included in the relevant/rest dataset. We compare the evaluation on the relevant/not dataset, when estimating the parameters with both the relevant/not (test A) and relevant/rest (test C), to determine which has higher performance when trying to separate relevant sentences from not-relevant sentences.

### Methods for objective 2: ranking articles by risk of bias

Objective 2 aims to rank articles by risk of bias, by training a logistic model to predict the risk-of-bias value of each article. The scores output by the model are used to rank articles by predicted risk of bias. In this section we also compare the performance when using just the title and abstract text retrieved from the PubMed database, rather than the full-text article. We generate models using: (i) the full-text content of the research articles; (ii) the article title from the PubMed database; and (iii) the article title and abstract from the PubMed database.

### Methods for objective 3: reducing the number of assessments the reviewers need to perform by hand

Objective 3 aims to reduce the reviewer work load by identifying articles that can be classified as low or not-low with high enough certainty, so that only a single reviewer is needed to assess these articles by hand. We suggest that the certainty is high enough when the model's assignment is at least as likely to be correct as an assignment by a human reviewer. When this is the case, it may be reasonable to replace a human reviewer by this model prediction. As already mentioned, the logistic regression model produces a well-calibrated score s for each article, such that *s* can be interpreted as the probability that the article has a risk of bias value of low. We can compare the scores assigned by a model to two fixed probability thresholds *t* and *1 − t* , where *t* is an estimate of the proportion of human assignments that are correct. Articles are classified as low for a property if *s* ≥ *t* and as not-low if *s ≤ 1* − *t.* This assumes that the human reviewer makes the same proportion of mistakes with not-low and low articles, respectively.

To determine the value of threshold *t* we use results of previous work by Lensen *et al.*^8^ and Hartling *et al.*^5^ These works analysed the degree of concordance of risk-of-bias assignments given by reviewers who have assessed the same studies. Using the binary labels low risk and high/unclear risk, Hartling *et al.*^5^ found disagreements (number of disagreement/number of comparisons) of 11/123, 26/123 and 41/123 for sequence generation, allocation concealment and blinding, respectively. The Cohen kappa values are 0.82, 0.31 and 0.30, respectively, such that higher agreement (relative to that expected by chance) is achieved for sequence generation compared with allocation concealment and blinding. Lensen *et al.*^8^ reported disagreements of 8/28, 19/46 and 20/31 for sequence generation, allocation concealment and blinding, respectively. We calculate the average proportion of disagreements across these studies and properties to give an estimate of the proportion of articles with reviewer disagreements of 26.4%. For these articles, we know that one assignment is incorrect and the other is correct, such that exactly half of the assignments for these reviews are incorrect. For the other article assignments where both reviewers agree (the remaining 73.6%), we cannot know whether they are both correct or both incorrect. We assume that if two reviewers agree, then they are both correct, such that all incorrect assignments are accounted for in the 26.4% of articles with disagreements. As exactly half of the assignments for articles with disagreements are incorrect, the proportion of assignments that are incorrect (under the above assumption) is 13.2%. Therefore, a probability that is higher than 0.868 would be better than the certainty of a human reviewer, and we set the threshold value *t* to 0.868.

The lower threshold, *1* − *t* = 0.132, denotes the score below which we are at least as certain as a human reviewer that an article has an assignment of not-low, according to the model prediction. The upper threshold, *t* = 0.868, denotes the score above which we are at least as certain as a human reviewer that an article has an assignment of low, according to the model prediction. A score between 0.132 and 0.868 indicates that the model could not predict the label with as much certainty as a human reviewer, and these articles should be assessed as usual by two reviewers.

## Illustrative Results

### Objective 1: identifying relevant sentences

**Figure 5.**
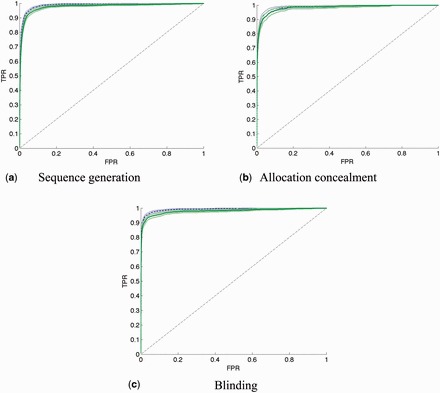
ROC curves for sentence-level learning comparing results using the relevant/not dataset, compared with the train relevant/rest, test relevant/not results, for each risk-of-bias property. Test B: blue dashed curve. Test C: green solid curve. Generated with parametric rate-oriented method ^33^ with associated point-wise confidence bounds. This method requires a constant number of examples of each label (low,not-low) in each fold, so we add examples to make N constant, and use random selection of examples to correct these frequencies, for instance by removing a randomly selected positive example and duplicating a randomly selected negative example in a particular fold. TPR: true positive rate; FPR: false positive rate.

Results of the comparisons of tests A, B and C are given in Table 2 , the average number of parameters in each model is given in Supplementary Table 1 (available as Supplementary data at *IJE* online) and the ROC curves of the models generated in test B and test C are shown in Figure 5 . These indicate very good ranking performance, as the ROC curves pass near to the point (0,1) in ROC space. We evaluate the models using the area under the ROC curve (AUC) metric, because for this objective we are concerned with how well our models are able to rank sentences by relevance. We are concerned with ranking rather than classification because we seek to provide an ordering to the reviewer such that they can see the most relevant sentences in an article. For example, the ranks allow sentences to be highlighted with different colours or shades in an electronic version of the article. The AUC evaluates ranking performance across all positions in a ranking. Previous text mining work has used metrics that focus only on a set of *n* highly ranked examples. ^18^^,^^25^ For this sentence ranking task, it is appropriate to assess the whole ranking because, when a reviewer views an article, they are not restricted to viewing only the top *n* sentences but can if they wish view all sentences. Hence, ranking a relevant sentence halfway down the ranking, for instance, rather than at the end of the ranking, still provides some benefit to the reviewer.

Table 2 gives the numbers of sentences that are relevant, not-relevant and unlabelled for each risk-of-bias property, and the results as the mean AUC across the 10 folds of cross-validation. We compare the results of tests B and C using a two-tailed paired t-test that compares the AUC evaluated on the models of the 10 folds. We use an unpaired t-test to compare the results of tests A and B because these tests evaluate the models with different sets of sentences.

The ranking performance of all three sentence level tests (A–C) across all three risk-of-bias properties was consistently high (all between AUC = 0.974 and AUC = 0.991). Training and evaluating models using the relevant/not data produced better performance compared with training and testing models using the relevant/rest dataset, for two of our three labels (tests A vs B in Table 2 ). This may be because the relevant/rest dataset is noisier as it has some relevant sentences labelled as rest rather than relevant. This can have two effects. First, it is more difficult for the model to separate the relevant examples from the rest examples. Second, when evaluating the test data, the relevant sentences that have been incorrectly labelled as rest would be evaluated incorrectly. Comparing the performance between training using the relevant/rest dataset and training using the relevant/not dataset, while testing both using the relevant/not labelling, we again found that the model trained with the relevant/not labelling method gave a better performance for two of the three properties (test B compared with test C).

These tests have indicated that the relevant/not labelling should be used to train models to predict sentence relevance. These models gave a very high ranking performance, with mean AUC values across the 10 folds higher than 0.985 for all three properties. This can be interpreted as follows. Given a randomly selected relevant sentence, and a randomly selected not-relevant sentence, the probability that the relevant sentence would be ranked more highly than the not-relevant sentence is higher than 0.985.

### Objective 2: ranking articles by risk of bias

We again use the AUC to evaluate the ranking performance of these models, given in Table 3 as the average AUC across the 10 folds of cross-validation. The AUC is an appropriate metric because it evaluates ranking performance across the whole ranking. A reviewer assesses all articles in a systematic review, such that ranking a low article before a not-low article is beneficial at any point in the ranking.

**Table 3.** Results for objective 2: ranking performance using different datasets and *P* -values comparing these models using a paired two-tailed t-test

Dataset	*seq-gen*	*alloc-conc*	*blind*
AUC (standard deviation)			
1. Article content	0.769 (0.051)	0.777 (0.034)	0.726 (0.051)
2. PubMed title	0.682 (0.053)	0.690 (0.072)	0675 (0.063)
3. PubMed title and abstract	0.692 (0.037)	0.685 (0.047)	0.694 (0.065)
*P* -values: ^1^ comparison of performance using feature sets 1, 2 and 3			
1 vs 2	0.001	0.004	<0.001
1 vs 3	<0.001	0.002	0.206
2 vs 3	0.672	0.741	0.497

^1^ *P* -values using two-tailed paired t-test to compare the AUC values across the 10 folds of cross validation.

**Figure 6.**
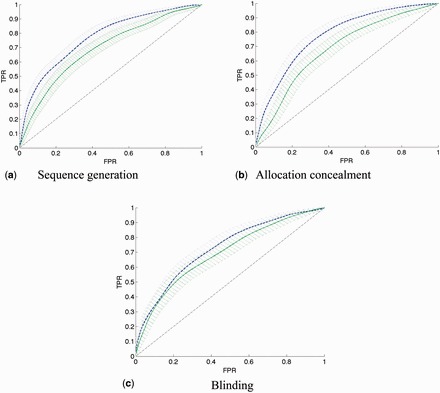
ROC curves for predicting article risk of bias. Generated with parametric rate-oriented method ^33^ with associated point-wise bounds. Blue dashed line: article contents (model 1); green solid line: PubMed title and abstract (model 3); TPR: true positive rate; FPR; false positive rate.

The mean [standard deviation (SD)] number of parameters across the 10 folds for each model is given in Supplementary Table 1 . The ROC curves of the models generated using the full text and the title and abstract only are shown in Figure 6 . The models using the full text had mean AUC > 0.72. The models using the PubMed title had mean AUC > 0.67. The models using the PubMed title and abstract had mean AUC > 0.68. All models are better than random (all permutation *P* -values < 0.001, which denotes the proportion of random models achieving an AUC higher than our model—see supplementary material S3 for details). Models using the article content are able to rank articles better than when using only the title, or title and abstract from the PubMed database, for the sequence generation and allocation concealment properties only. We did not find a difference between using the title and using the title and abstract, although this may be due to a lack of power because our sample size is small.

### Objective 3: reducing the number of assessments the reviewers need to perform by hand

Table 4 shows the number of articles our models classify as low or not-low using these score thresholds. We check the model calibration (see supplementary material S4 , available as Supplementary data at *IJE* online) and find, as expected, our models are well calibrated. All models were able to classify more than 33% of articles as either low or not-low with a certainty at least as high as a manual reviewer. We suggest that only one human reviewer is needed to assess these articles manually.

**Table 4.** Results for objective 3: mean number of articles (standard deviation) and precision (standard deviation) across 10 folds (using sentence model)

	Predicting not-low		Predicting low		
	score ≤ 0.132		Score ≥ 0.868		
	% articles	Precision	% articles	Precision	Total %
*seq-gen*	16.9 (2.42)	0.821 (0.114)	20.9 (2.77)	0.838 (0.072)	38.2 (3.14)
*alloc-conc*	15.5 (2.92)	0.874 (0.090)	9.9 (2.77)	0.816 (0.181)	35.5 (4.45)
*blind*	7.6 (2.91)	0.803 (0.145)	14.8 (2.62)	0.810 (0.101)	33.4 (6.58)

## Discussion

We have identified and addressed three key objectives to assist the risk-of-bias assessment process using an automated text mining approach. We have shown that we can rank sentences by predicted relevance (for each risk-of-bias property) with high ranking performance (AUC > 0.98). This is useful to assist reviewers by indicating which parts of the article text are particularly relevant to risk of bias. We were able to rank articles according to risk of bias with AUC > 0.72. Ranking articles by risk of bias means that the reviewer is able to assess the articles from predicted low to predicted not-low risk of bias. A web-based prototype to demonstrate our methods can be found at [ http://www.datamining.org.uk/sysreview.html ].

We found a small decrease in performance when using only the article title and abstract from PubMed, compared with using the full text extracted from the article PDF document for sequence generation and allocation concealment (t-test *P* < 0.001 and *P* = 0.002, respectively). The full-text content will often contain more information about risk of bias compared with the title and abstract alone. However, this benefit may be offset by noise from extraction of the article content from PDF documents (due to the fact that text in PDF documents is stored as a set of character locations which then need to be processed to form the word, sentence and paragraph structures of the original document). Also, much of the content in the full-text article is likely to be irrelevant to risk of bias. Conversely, whereas the PubMed title and abstract may not contain as much information about risk of bias compared with the full text, it may have less noise because this text is a concise summary of the full-text article retrieved from the PubMed database. Retrieving PubMed data is quick and straightforward, whereas obtaining the full text of research articles requires more effort, and text extractions from PDF documents are noisy. Hence, the increase in performance from using the full text may not be worth the cost of retrieving the article full text.

The PubMed title was predictive of risk of bias, with AUC > 0.67 for all three properties. Although the number of features was small (circa 60–100), the PubMed title contains a set of key terms describing the study and often contains terms such as ‘blind’ and ‘randomized’ that are relevant to risk of bias. For instance, if a title includes the term ‘double-blind’ then we would expect the study to be more likely to have a low risk of bias compared with those studies that do not have this term in the article title. Indeed, 77% of the articles with ‘double blind’ in the title are assigned a low value (76 low vs 23 not-low).

Our results indicate that it is possible to use text mining to reduce the reviewer workload, by identifying the articles that have been classified with a certainty higher than that of human reviewers. We suggest that these articles only need to be manually assessed by one reviewer. On average more than 33% of research articles can be labelled as low or not-low with higher certainty than that of a human reviewer, offering the potential to reduce the amount of time required by human reviewers. We note that if the class distribution changes between the data used to train a model and the data on which scores are predicted, a score adjustment is needed before this classification into low and not-low is used (discussed in more detail in supplementary material S5 , available as Supplementary data at *IJE* online).

Our work has the following limitations. First, the limited size of our dataset (between 671 and 989 per risk-of-bias property) may have restricted the performance of our models. Also, this meant that the performance of the article-level models could not be estimated precisely (as shown by the wide confidence intervals around the ROC curves). This limited our ability to test more complex models, because it would be unlikely that any difference in performance (compared with our simple approach) could be detected using this small dataset. Second, we only included articles if the title and abstract could be found in the text extracted from the PDF articles, such that articles with poor text extractions are less likely to be included in our dataset because noise within the text means that the title and abstract may not be found. Therefore, it is likely that our dataset is less noisy than study articles on average. Furthermore, we only include articles in our dataset where a quotation was supplied or no information was stated, and so it is possible this sample is unrepresentative of articles describing clinical trials. Third, we use labels inferred from data from Cochrane risk-of-bias assessments such that these labels may not be the same as directly annotated labels. Last, previous work has indicated discordance between reviewers who assess the same article, and this indicates that the labels we have used from the Cochrane risk-of-bias assessments may not always be correct.

An automated approach is limited by the degree of reporting in trial publications, as although the CONSORT statement specifies that information relevant to risk of bias should be described in a trial report, this is often not the case. ^31^ However, it is known that trial protocols can contain information that is not reported in the study publications, ^32^ hence risk-of-bias information could potentially be extracted from these protocols.

To our knowledge, only recent work by a single research group has investigated text mining for risk-of-bias assessments. ^11^^,^^23–25^ Similarly to our work, Marshall *et al.* aim to predict the risk-of-bias value described in research articles and to identify relevant sentences within these articles, using linear models. Our work differs from this work in the following ways. First, a key aspect of our work was to identify and tackle three concise objectives that use machine learning to practically support risk-of-bias assessments. We evaluate our models using metrics that are appropriate for each of these objectives. Second, although we use a different subset of the same corpus, our sentence labelling uses sentences known to have a not-relevant label (because the article has been described as containing no information), and we found our sentence labelling approach gave an improved performance. Last, our work includes an experimental comparison of performance using the full text compared with using only the title and abstract from the PubMed database.

We believe this work takes an important step towards assisting risk-of-bias assessments using machine learning methods. The performance of our models is encouraging, but could be improved further by creating a larger dataset with which to train the model parameters. Furthermore, future work should involve user testing to evaluate these assistive techniques in a practical setting.

## Funding

This work was supported by a UK Medical Research Council studentship to L.A.C.M. This work was also supported by Medical Research Council grant MC_UU_12013/1‐9.

## Supplementary Material

Supplementary Data
