# Supplementary material for: Machine learning to assist risk-of-bias assessments in systematic reviews
Source: Int J Epidemiol. 2015 Dec 8;45(1):266–77. doi: 10.1093/ije/dyv306 (PMC4795562; doi:10.1093/ije/dyv306)
Supplement: Supplementary Data [file dyv306_supplementary_data.zip › ije-2015-03-0411-File002.pdf]

Machine learning to assist risk of bias assessments in systematic reviews

Louise A.C Millard* [1,2,3], Peter A Flach [1,3] and Julian P.T Higgins [1,2]

* Corresponding author. Email: louise.millard@bristol.ac.uk

[1] MRC Integrative Epidemiology Unit, University of Bristol, United Kingdom

[2] School of Social and Community Medicine, University of Bristol, United Kingdom

[3] Intelligent Systems Laboratory, University of Bristol, United Kingdom

Supplementary material

S1 Creating the dataset

We created a dataset collected using risk of bias assessments from Cochrane reviews [1]. This provides values of the risk of bias properties of a set of studies. The review stores the data entered by the reviewers as XML files. Each XML file contains the risk of bias assessments for studies of a single systematic review. This information includes a *yes*, *no* or *unknown* value given to each risk of bias property, for each particular study. Assigning the value *blinding=low*, for instance, means that the blinding carried out was adequate for this study such that the risk of bias is low. The *yes*, *no* and *unknown* values correspond to *low*, *high* or *unknown* risk of bias respectively.

The XML files also include the references of the articles that the reviewer assessed for each study. Thus we have a set of studies, with a corresponding set of references, and risk of bias judgements. An example is shown in Figure 2 in the main article. However, we have no direct mapping between a reference and a risk of bias judgement. There may be a number of references attached to a study but only one, for instance, may describe the method of sequence generation. Alternatively, this property could have been determined from another source, and may not be described in any of the referenced articles. Alternative sources include the study protocol or direct communication with the study researchers.

To infer a direct mapping between references and risk of bias assignments we use information in a descriptions field that is attached to each risk of bias assignment. Reviewers use this field to provide evidence justifying their risk of bias assignment. It is common that reviewers will do this by simply entering a quotation of the relevant text from a research article. If we can find the quotation for a property within an article then this maps this article to this risk of bias property value. The example in Figure 2 shows a study with a quotation for the blinding property, which is found in the article content of reference 2. The article of reference 2 can therefore be used in our dataset with a labelling for blinding only.

We extract the quotations from the descriptions field by identifying single and double quotation marks, using regular expressions. We also checked for the case where a reviewer

had entered an incomplete quotation using the '. . . ' notation. We dealt with this by saving the text either side of the '. . . ' as separate quotations, such that when labelling using these two quotations the same sentence would be labelled using these.

Reviewers often used the term 'no information' or variants of this, to indicate that they could not find any relevant information in the research articles for a particular risk of bias property. Any research article describing this study can then be labelled with the value given for this property, as we can infer that the lack of information is the reason for this choice of label value. For instance, an article may have the label *unknown* for blinding and 'no information' in the description field because all research articles referenced for this study in this review have been found to contain no relevant text, such that an assignment for the property value to *low* or *high* could not be given.

In this work we use the full text content of research articles. We retrieve a set of articles by finding the page for this article on the PubMed website corresponding to the references from the Cochrane data. We restrict only to the references of studies where a quotation or no information was reported in the Cochrane risk of bias tool data. We locate the full text article using the links available on the PubMed article page. We then ensure that the PubMed title and abstract could be found in the extracted text of this article. Where a quotation is supplied, we only include this article in the dataset, for this risk of bias property, if this quotation is found in the article text. Where no information is indicated, we include this article in our dataset for this risk of bias property.

We segment the article text into sentences (using Stanford's Natural Language Processing parser). We parse each sentence and search for the quotations attached to the study, and store the article as a set of sentences, with quotation information that relates a particular sentence to a quotation.

	Sentence models			Document models		
	A	B	C	All	Title only	Title and abstract
seq-gen	14845.6 (26.49)	2372.2 (9.95)	14845.6 (26.49)	12176.7 (50.98)	98.9 (4.25)	1463.2 (7.60)
alloc-conc	12059.1 (30.31)	5539.4 (18.50)	12059.1 (30.31)	9907.6 (53.97)	60.1 (2.47)	1144.3 (13.31)
blind	11408.7 (33.28)	3004.2 (9.09)	11408.7 (33.28)	9352.9 (26.84)	64.5 (2.01)	1090.8 (8.16)

Table 1: Mean number of features across cross-validation folds (standard deviation)

S2 Using unregularised regression

Regularisation is often applied to text mining tasks because these commonly have a large number of features such that it is easy for the model to overfit to the training data. We attempted using logistic regression with regularisation for this work using the glmnet R package. We used ridge regression by setting the parameter $\alpha = 0$, and use the cv.glmnet function in order to learn the lambda parameter. We learnt the lambda parameter (denoting the degree of regularisation) using 3-fold cross-validation on the training data. This means that for each 90% used to train the model this was divided into three folds used to choose the lambda value. The lambda giving the lowest mean AUC across the three folds was then chosen to train the model using the whole training set (90% of the dataset) which was then evaluated on a given fold (10% of the dataset).

Using this method we found that often the models would output a constant value, the class distribution of the training data, such that the classes cannot be distinguished. This may be because the lambda chosen was too high such that the model parameters were too constrained. This inability to choose an appropriate lambda value is likely to be due to the small size of our dataset.

S3 Comparing AUC of models with AUC of random models

We use permutation testing, where we take the true labels of the original cross validation test folds, and randomly permute the order in each fold to give a random ranking with the same number of positives and negatives. We calculate the average AUC of these 10 test folds. This is repeated 1000 times and we give the proportion of times that an AUC greater than that of our models is found with these random rankings.

S4 Calibration maps

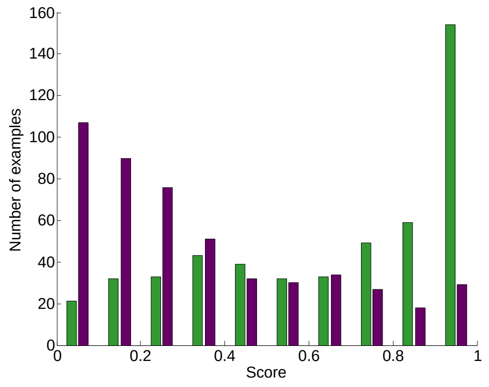

(a) Sequence generation

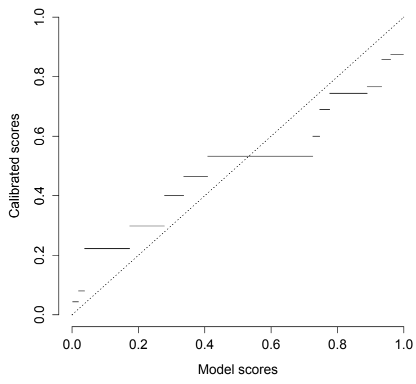

(b) Sequence generation

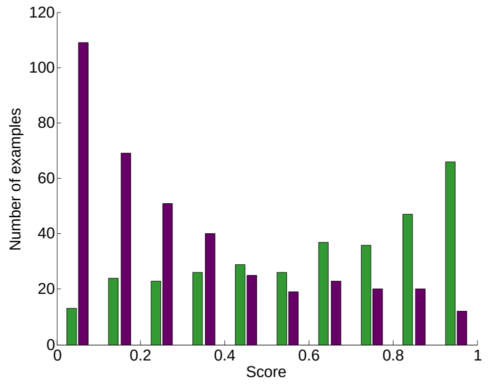

(c) Allocation concealment

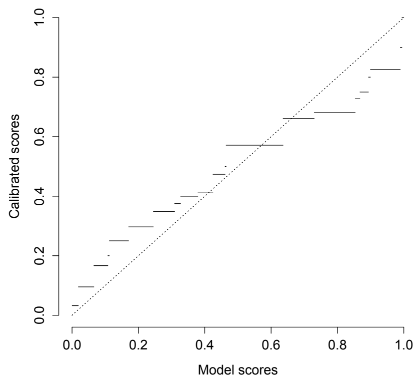

(d) Allocation concealment

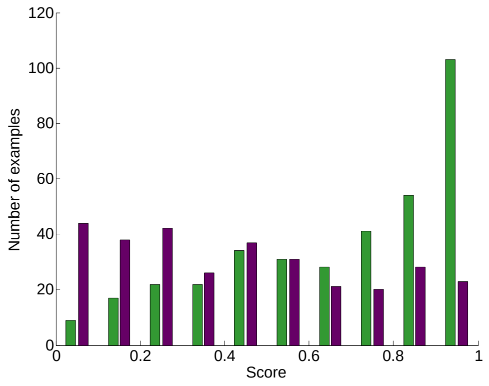

(e) Blinding

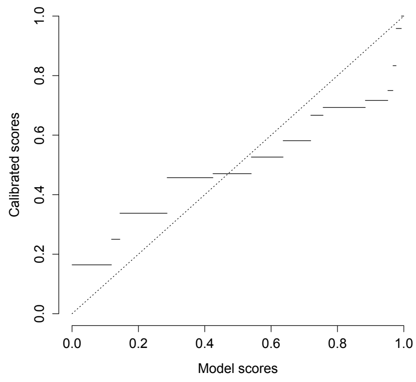

(f) Blinding

Figure 1: Left: Score distributions predicted by logistic regression models generated with cross-validation (green: *low*; purple: *not-low*). Right: Assessing calibration with reliability diagrams [2] (generated with isotonic regression). We used the CORElearn R package to perform isotonic calibration to create the reliability diagrams using the scores output from the 10 cross validation models of each property.

S5 The effect of changes in the proportion of *low* and *not-low* articles when predicting risk of bias from research articles

Logistic regression is known to produce scores that are well calibrated. Scores are calibrated if, for example, given a set of examples that all have a score of 0.8, we can expect 80% of these examples to have a label of *low* (assuming a high score denotes more likely to be *low*). This means that we can use these scores as probabilities that an example belongs to a particular class [3, 4].

The scores output by a model are only calibrated if the proportion of *low* and *not-low* articles remains constant between the data used to train the model, and the new data on which predictions are made. The reason can be shown with an example. Given a set of 10 articles all with score 0.8. We would expect that 8 have *low* risk of bias and 2 have *not-low* risk of bias. Now imagine duplicating the positive examples, such that we have 18 articles. As these are duplicates the model would again output 0.8 for all 18 articles. However, now there are 16 *low* articles and 2 *not-low* articles, so the proportion of positives is $\frac{8}{9}$ and the scores are no longer calibrated.

While our dataset has balanced classes, such that approximately equal number of articles are labelled as *low* and *not-low* risk of bias, in reality the classes are not balanced, with larger numbers of *not-low* articles compared to *low* articles (see Table 1). This means that our article level classifiers will not be calibrated when applied in future. We can adapt our classifiers in order to recalibrate them using a simple adjustment of scores output by these models:

$$s' = \frac{s \cdot scalar_{pos}}{s \cdot scalar_{pos} + (1 - s) \cdot scalar_{neg}} \quad (1)$$

where:

$$scalar_{pos} = \frac{\pi'}{\pi} \quad (2) \qquad scalar_{neg} = \frac{1 - \pi'}{1 - \pi} \quad (3)$$

where π is the proportion of positives in the data and the new data is denoted by $'$.

For example, we can use this to adjust for the change of class distribution for $s = \frac{8}{10}$

in the example above, where $scalar_{neg} = \frac{2}{18} \div \frac{2}{10} = \frac{5}{9}$ and $scalar_{pos} = \frac{16}{18} \div \frac{8}{10} = \frac{10}{9}$:

$$\begin{aligned} s' =& \frac{s \cdot scalar_{pos}}{s \cdot scalar_{pos} + (1-s) \cdot scalar_{neg}} \\ =& \frac{\frac{8}{10} \cdot \frac{10}{9}}{\frac{8}{10} \cdot \frac{10}{9} + (1 - \frac{8}{10}) \cdot \frac{5}{9}} = \frac{8}{9} \end{aligned} \tag{4}$$

This adjusted score corresponds to the correct probability after the change of class distribution.

In objective 3 we use two score thresholds at $s = 0.868$ and $s = 0.132$. These denote the probabilities above and below which we are at least as sure as a human reviewer that the prediction made by our model is correct. These thresholds are fixed across all class distributions. This means that when adapting the calibration to new data we can expect less scores assigned above the upper threshold (predicting *low*, at s=0.868) and more scores assigned below the lower threshold (predicting *not-low*, at s=0.132). This is because the new data has a lower proportions of positives (with higher score on average) and a higher proportions of negatives (with lower score on average) than the data on which the models were trained.

References

[1] Higgins JPT, Altman DG, Gøtzsche PC, Jüni P, Moher D, Oxman AD, et al. The Cochrane Collaborations tool for assessing risk of bias in randomised trials. BMJ. 2011;343.

[2] Zadrozny B, Elkan C. Obtaining calibrated probability estimates from decision trees and naive Bayesian classifiers. In: ICML. vol. 1. Citeseer; 2001. p. 609–616.

[3] Niculescu-Mizil A, Caruana R. Predicting good probabilities with supervised learning. In: Proceedings of the 22nd International Conference on Machine Learning. ACM; 2005. p. 625–632.

[4] Flach PA. ROC analysis. In: Encyclopedia of Machine Learning. Springer; 2010. p. 869–875.
